# Factors Influencing Willingness to Be Vaccinated against COVID-19 in Nigeria

**DOI:** 10.3390/ijerph19116816

**Published:** 2022-06-02

**Authors:** Abayomi Samuel Oyekale

**Affiliations:** Department of Agricultural Economic and Extension, North-West University Mafikeng Campus, Mmabatho 2735, South Africa; abayomi.oyekale@nwu.ac.za

**Keywords:** COVID-19, vaccines, coronavirus, acceptance, Nigeria

## Abstract

Vaccines have been historically used to address some pressing health problems in the world. COVID-19 presents no exception, although vaccine hesitancy remains a major bottleneck in some countries. This study analyzed the factors influencing willingness to be vaccinated against COVID-19 in Nigeria. The data are from the 10th wave of COVID-19 National Longitudinal Phone Survey (COVID-19 NLPS) that was conducted in 2021. The data were analyzed with Logit regression. The result showed that the national acceptance rate of COVID-19 vaccine was 85.29%. The North East had the highest acceptance rate (96.14%), while the South East (71.80%) had the lowest value. Rural areas had higher vaccine acceptance rates of 87.80% as compared against 81.41% for urban areas. Logit regression result showed that urban residents in the South East zone and the South South zone were not too worried about contracting COVID-19 or not worried at all about contracting COVID-19, saw COVID-19 as not much of a threat to household finance or COVID-19 as not a threat at all to households’ finances, which significantly reduced the log odds of residents’ willingness to be vaccinated against COVID-19 (*p* < 0.05). However, age, the COVID-19 preventive compliance index and needing medical services significantly increased the log odds of the willingness to be vaccinated against COVID-19 vaccines (*p* < 0.05). Addressing the COVID-19 pandemic in Nigeria through vaccination requires significant interventions for ensuring regional and sectoral balances in vaccine acceptability through interventions and programmes for promoting individuals’ perception of health risk and vulnerability.

## 1. Introduction

The twenty-first century is witnessing a global public health crisis in the form of flu-like infection that is caused by a coronavirus. This pandemic comes with some perplexing and traumatic impacts, causing health hazards of heartbreaking significance. Christened by the World Health Organization (WHO) as COVID-19 on the 11th February 2020, the disease still remains a major development hurdle and public health concern in many developed and developing countries [1]. More than two years after its discovery, COVID-19 still constitutes a devastating health problem with significant morbidity and mortality consequences [2]. More importantly, the pandemic is a candid reflection of Africa’s vulnerability to global health hazards due to economic reintegration and globalization [3].

The COVID-19 pandemic affected the Nigerian economy because of a global decline in oil prices [4]. The pandemic was another form of unprecedented economic shock that came barely four years after the country escaped from economic depression [5]. After 25 years of steady economic growth, the recession between 2014 and 2015 resulted in the contraction of Nigeria’s gross domestic product (GDP) by 1.6% [5]. Moreover, available statistics revealed that in 2020, the Nigerian economy shrank by 1.8%, which was the highest negative growth rate in the GDP since 1983 [6]. To be precise, the gross domestic product (GDP) declined by USD 11 billion (23%) within the first eight weeks of national lockdown [7]. The COVID-19 pandemic is now hurting Nigeria’s economic recovery, driving slow economic growth, unemployment and inflation [8].

The provision of a lasting solution to this pandemic is therefore a collective goal that had been pursued by international policy makers and stakeholders in the health sector. The World Health Organization (WHO) has advocated for mass vaccination, to reduce associated morbidity and mortality from the pandemic. This is commendable because vaccines are notable components of effective healthcare service delivery, and they are able to enhance the immune system through the production of antibodies [9,10,11]. More importantly, the contributions of vaccines in the elimination of previously infectious diseases across different regions of the world cannot be overemphasized [9,10,12]. Similarly, the antecedents of vaccine hesitancy in different parts of the world had been acknowledged [13,14].

In Nigeria, the non-availability of vaccines initially slowed the pace of addressing the COVID-19 pandemic. It was also not certain if majority of the population would be willing to be voluntarily vaccinated. This was due to COVID-19-related misinformation making the rounds on some social media platforms [15,16,17]. Historical records of vaccine hesitancy in Nigeria also raised some concerns. For instance, the polio vaccine was boycotted in northern Nigeria, based on speculations of its adverse side effects [18]. The procured COVID-19 vaccines in Nigeria are AstraZeneca, Moderna, Pfizer and Johnson & Johnson [19]. The slow pace of the COVID-19 vaccine turnout and acceptability in Nigeria is reflected by the fact that only about six million people received the first dose as of 19 November 2021, while 3,369,628 had already taken the second dose [20]. As of 18 February 2022, more than 20 million doses of the vaccines had been administered [19], and approximately 44 million had been administered as of 27 May 2022 [21]. Acceptance rates of COVID-19 vaccines in Nigeria among the adult population vary based on demographic characteristics and geopolitical zones and are between 20.0% to 58.2% [22].

An individual’s perception of health risks can be associated with the decision to comply with disease preventive behavior like vaccination. The perception of health risks and the decision to be vaccinated are often interrelated [23]. Compliance with preventive measures is possible when individuals can properly evaluate their levels of health risk [24]. This is the heart of behavior change models such as the subjective expected utility theory [25,26,27], the health belief model [28], the theory of reasoned action [29] and the theory of planned behavior [30]. Some authors found that perception of health risks that are associated with COVID-19 increased the willingness to be vaccinated in the United States of America (USA) [31], Germany [32] and France [33], while it reduced it among some Australian respondents [34]. Moreover, of concurrent importance is vaccine safety [35,36], which appears to be the bottom line for ongoing vaccine hesitancy. Specifically, COVID-19 safety concerns are diverse and most of them are related with immediate side effects and effectiveness [37].

In some other studies, individuals’ demographic and socioeconomic characteristics influenced COVID-19 vaccine hesitancy. However, in terms of the directions of influence, different results were obtained. Specifically, old age is a notable risk factor of COVID-19 infection and intention to be vaccinated [38,39,40,41]. Some studies have reported a high level of hesitancy among older people [42,43,44,45]. Other studies found education level to have some positive influences on the willingness to be vaccinated against COVID-19 [36,41,42,43]. Significant associations between vaccine hesitancy and gender had been reported by some studies with the conclusion that male respondents had a higher likelihood of being vaccinated against COVID-19 vaccines [36,41,46,47]. Being married was reported to increase vaccine acceptability in Nigeria [45], although a contrary finding was reported by a study in Uganda [44].

Some households’ socioeconomic variables were explored in relation to vaccine hesitancy. Nikolovski et al. [36] found that the willingness to be vaccinated was significantly associated with the income levels of households. Similar findings had been reported by Wake [41]. In another study, respondents who were not earning salaries had a significantly higher willingness to receive a COVID-19 vaccination [44]. Some other studies found that health-insured individuals had a lower probability of accepting a COVID-19 vaccine [40], while confidence in health systems increased the probability of vaccine acceptance [42]. Some studies analyzed the impact underlying health conditions like cardiovascular diseases and diabetes. It was found that obesity and the presence of chronic diseases reduced the willingness to be vaccinated against COVID-19 [40]. Additionally, from an occupational point of view, farmers and those residing in rural areas had low probability of being vaccinated against COVID-19 [42].

The reviewed studies have highlighted differences in the effect of selected explanatory variables on vaccine hesitancy across different countries. This is a reflection of existing national and regional diversities in individuals’ behavioral attitudes towards the acceptability of COVID-19 vaccines. This study therefore analyzed the determinants of COVID-19 vaccine hesitancy in Nigeria using a highly representative dataset. More importantly, Nigeria’s rollout of COVID-19 vaccination has been very slow, and this study provides some policy-prompting insights that could guide health policy makers in their quest towards the promotion of the effective management of COVID-19 through mass vaccination and other prevention protocols. This is needed to prevent unnecessary medical emergencies caused by the exponential increase in positive COVID-19 cases in a country of more than 200 million people with a deficient healthcare delivery system and significant cultural, regional, sectoral and social development diversity [48].

## 2. Materials and Methods

### 2.1. The Data and Sampling Procedure

The study used the data from the Nigeria’s COVID-19 National Longitudinal Phone Surveys (COVID-19 NLPS) [49]. The data were collected through telephone interviews. The surveys used the sampling frame of the fourth wave of the 2018/2019 General Households Survey (GHS) panel data. This sampling frame comprised of 4976 households, and majority of them (4934) provided their mobile telephone numbers. Although a sample size of 1800 was initially targeted, 3000 households were selected for interviews to cater for expected high non-response rate as is always the case in telephone interviews.

The data were collected as monthly panel surveys. During the first wave, 1950 households successfully completed the survey out of 3000 that were contacted in April/May 2020. These are the households that formed the baseline database for subsequent monthly surveys. During the second wave, 1820 households were successfully interviewed in June 2020. During tenth wave, 1700 households were successfully interviewed in February 2021. This study used the tenth wave of the surveys because of its comprehensive coverage of COVID-19 vaccination intentions. The question on vaccination intention, which was analyzed in this study was phrased in the questionnaire as: If an approved vaccine to prevent coronavirus was available right now at no cost, would you agree to be vaccinated? It should also be noted that some relevant demographic characteristics of the household heads, such as education and gender that were missing in the tenth wave data files were obtained from previous surveys.

Sample weights were provided at every round of the data collection to ensure representativeness of the selected samples. The phone interviews were carried out by National Bureau of Statistics (NBS) interviewers who were properly trained virtually. The enumerators made phone calls to the selected households, and interviews were carried out with a representative member, preferably the head of the household or someone with proper knowledge of issues within the household. This individual is expected to be contacted in future surveys, although another authorized representative can respond if the previous respondent is absent.

### 2.2. Estimated Logit Regression Model

Logit regression model use the maximum likelihood estimation method to fit a binary or ordinal dependent variable [50,51]. In this study, the dependent variable is binary in nature, coded 1 for willingness to be vaccinated and 0 otherwise. Therefore, given a dependent variable $y_{i}$ that denotes the response of an individual *i* on their willingness to be vaccinated against COVID-19, based on a set of explanatory variables ($x_{1i}\ldots \ldots x_{ki})$, logistic regression can be used to find the probability distribution for this individual. This is specified in Equation (1).

(1) $$P\left(Y=1/x_{1}\ldots \ldots \ldots \ldots x_{k}\right)=f\left(x_{1}\ldots \ldots \ldots \ldots x_{k}\right)$$

Equation (1) can be written as a logistic distribution function:

(2) $$\left(Y=1/x_{1}\ldots \ldots \ldots \ldots x_{k}\right)=\frac{\exp\left(\beta_{0}+\beta_{1}x_{i}+\cdots \ldots \ldots \beta_{n}x_{n}\right)}{1-\exp\left(\beta_{0}+\beta_{1}x_{i}+\cdots \ldots \ldots \beta_{n}x_{n}\right)}$$

Equation (2) can also be written as:

(3) $$ogitP\left(Y=1/x_{1}\ldots \ldots \ldots \ldots x_{k}\right)=\beta_{0}+\beta_{1}x_{i}+\cdots \ldots \ldots \beta_{n}x_{n}$$

The specifications of the dependent variable (Y) and independent variables (*x*) are contained in Table 1. The vector of the estimated parameters of the explanatory variables is denoted as 𝛽.

## 3. Results

The results in this section of the paper were generated from the 10th round of COVID-19 National Longitudinal Phone Survey (COVID-19 NLPS) that was conducted from the 6th to 22nd of February in 2020–2021. Table 2 shows that 85.29% of the respondents were willing to be vaccinated against COVID-19 in the combined dataset, while respondents from northern parts of Nigeria generally showed a higher level of willingness. Specifically, the North East zone had the highest proportion (96.14%) of residents being willing to be vaccinated against COVID-19 vaccines, while the lowest was from the South East zone (71.80%). Across rural–urban areas, residents in rural areas had a higher willingness to be vaccinated with 87.80%, compared to 81.41% in urban areas.

Table 2 also shows that 89.31% and 80.00% of the respondents with no formal education and tertiary education would be willing to be vaccinated against COVID-19, respectively. Additionally, 86.36% of the male respondents were willing to be vaccinated, as compared to 80.46% for females. Although the highest percentage (87.01) of the respondents that were willing to be vaccinated was in the 50 < 60 years age group, respondents who were 60 year or older had the lowest percentage of being willing to be vaccinated.

The results in Table 2 further showed that 86.43% of those who washed their hands after being to public places in a week preceding the interview were willing to be vaccinated as compared to 81.64% for those who did not wash their hands. Furthermore, 85.62% of the respondents who wore face masks while in public places were willing to be vaccinated as compared to 83.98% for those who did not wear masks. The need for medical services was registered by 743 respondents with 89.64% of them willing to be vaccinated.

Table 3 shows the distribution of the respondents based on being worried about households’ members contracting COVID-19. It shows that majority of the respondents were very worried. This is followed by those who were not worried at all. The results also show that the proportions of those who were willing to be vaccinated against COVID-19 vaccines are higher for those who were very worried and somewhat worried. Those who were not too worried or worried at all reported high proportions not willing to be vaccinated. The table further shows the distribution of the respondents based on their perceptions of the level of threat that COVID-19 places on households’ finances and willingness to be vaccinated. It shows that majority of the respondents indicated that COVID-19 portends a substantial threat to households’ finance. Additionally, willingness to be vaccinated also increased with perception of COVID-19 as a threat to the households’ finances.

The results of the Logit regression analysis are presented in Table 4. The model produced a good fit for the data given that the likelihood ratio chi-square (191.40) shows statistical significance (*p* < 0.01). The model was also tested for the presence of multicollinearity. The computed variance inflation factor (VIF) is 1.41 and it reveals that the model does not suffer from multicollinearity problem. Many of the included variables also show statistical significance.

Specifically, the parameter of urban residents is statistically significant (*p* < 0.05) with a negative sign. This implies that compared to the residents in rural areas, and holding other variables constant, residents in urban areas reduce the log odds of willing to be vaccinated against COVID-19 by a unit of 0.351. The computed odds ratio for the urban variable is 0.704. This shows that the odds of being vaccinated against COVID-19 for urban residents is 0.704 times that of rural residents. The parameter of the COVID-19 prevention compliance index is also statistically significant (*p* < 0.05). The results show that, holding other variables constant, a unit increase in the compliance index increases the log odds of being vaccinated against COVID-19 by a unit of 0.130. The odds ratio parameter also indicates that the odds of accepting a COVID-19 vaccine increases by 1.139 times when the compliance index increases by one unit.

The parameter of needing medical services has a positive sign and is statistically significant (*p* < 0.05). This shows that, holding all other variables constant, respondents that who needed medical services had the log odds of being vaccinated against COVID-19 that were higher by a unit of 0.310. Similarly, the odds ratio parameter shows that the odds of being vaccinated against COVID-19 for those who needed medical services were 1.376 times that of those that did not need medical services.

The parameter of gender had a positive sign but was statistically insignificant (*p* > 0.05). The parameter of the age of the household heads was positive parameter that was statistically significant (*p* < 0.05). This implies that as age increases by one year, the log odds of being vaccinated against COVID-19 increased by a unit of 0.014. Moreover, increase in age by one year increases the odds of being vaccinated against COVID-19 by 1.014 times. Although all the education parameters are statistically insignificant (*p* > 0.05), it should be noted that secondary education and vocational education had positive parameters as compared to negative parameters of primary and tertiary education levels.

Out of the zonal variables in the model, the North West, South East and South South showed statistical significance (*p* < 0.05) with positive, negative and negative signs, respectively. These results show that, compared to those in North Central, residents from the South East and South South zones had lower log odds of being vaccinated against COVID-19 by 1.092 and 0.831 units, respectively. However, compared to those from the North Central zone and, keeping other factors constant, residents from the North East zone had higher log odds of being vaccinated against COVID-19 by a unit of 0.848. In a like manner, the odds of being vaccinated against COVID-19 for residents in South East and South South zones are 0.336 and 0.436 times that for residents in north central zone. The computed odds ratio for the North East variable shows that the odds of being vaccinated against COVID-19 for residents in the North East is 2.334 times that of residents from North Central zone.

Some of the COVID-19 risk perception variables also showed statistical significance. Specifically, the results showed that the parameters of not being too worried about having COVID-19 and not being worried at all about having COVID-19 have a negative sign and are statistically significant (*p* < 0.05). These parameters imply that, compared to those who were very worried that their household members or themselves may contract COVID-19, respondents who were not too worried and not worried at all had the log odds of being vaccinated against COVID-19 reduced by 0.489 and 0.757 units, respectively. The computed odds ratios also show that the odds of being vaccinated against COVID-19 for respondents who were not too worried and not worried at all of getting sick with COVID-19 were 0.613 and 0.469 times that of those who were very worried.

Similarly, two of the variables that captured perception of the financial threats posed by COVID-19 showed statistical significance (*p* < 0.01). These results showed that compared to those who perceived COVID-19 as a substantial threat to their finances, those who indicated it was not much of a threat and not a threat at all had the log odds of being vaccinated against COVID-19 reduced by 0.652 and 0.686 units, respectively. The computed odds ratios also revealed that the odds of being vaccinated against COVID-19 for respondents who indicated that COVID-19 was not much of a threat and not a threat at all to their finances were 0.521 and 0.504 times that of those who indicated it to be a substantial threat.

## 4. Discussion

Average willingness to be vaccinated against COVID-19 was quite high (85.29%) in Nigeria when compared with results from other countries. Specifically, a study by CDC [52] found that 79% of people from Africa would be vaccinated against COVID-19 if they consider it safe. It was however noted that significant variations exist in willingness to be vaccinated against COVID-19, with Ethiopia and Niger having 94% and 93%, respectively, while Senegal and the Democratic Republic of Congo reported 65% and 59%, respectively. In a web-based survey that was carried out in Nigeria, it was found that the acceptance rate of the COVID-19 vaccine was 50.20% [47]. In another study [53], the acceptance rate of COVID-19 vaccines was between 29.4% and 86%. [54,55,56,57,58], while Echoru et al. [48] reported 53.6% for Uganda. Moreover, it should be noted that in some instances, willingness to undertake a behavior may be quite different from actual performance due to the interplay of socio-cultural and economic factors over time. Loomba et al. [59] reported that willingness to be vaccinated against COVID-19 cannot be taken as a static phenomenon. This is due to responsiveness of actual behavior to the emerging trail of vaccine misinformation and sentimental risk perceptions. However, Gibbons et al. [60] submitted that the intention to undertake a behavior is sometimes correlated with actual behavior.

The results also showed that respondents from northern Nigeria had higher odds of accepting COVID-19 vaccines. This finding is contrary to that of Adebisi et al. [61], who reported significantly higher odds of COVID-19 vaccine acceptance in southern Nigeria. Ordinarily, one would have expected lower vaccine acceptance in northern Nigeria because there have been historical boycotts of vaccination programmes in the region [18,62,63]. However, the global visibility of COVID-19, in terms of morbidity and mortality may have enhanced the risk perception of the northern population, the majority of which are illiterate. Similarly, COVID-19 prevalence statistics show that three northern states (Federal Capital Territory, Kaduna and Plateau) are among the top five states with the highest numbers of positive cases in Nigeria [64]. It should also be noted that at the onset of the COVID-19 pandemic about 1000 mysterious deaths were reported in Kano, the most populous state in northern Nigeria [65].

The proportion of rural dwellers that would be vaccinated against COVID-19 is higher than those from urban areas. Urban residents therefore have significantly lower odds of being vaccinated against COVID-19. Although urban residents are aware of the health benefits of vaccination, several conspiracy theories that are disseminated through social media about COVID-19 and the developed vaccines could have hindered the acceptability of the vaccines. This is a very critical concern because urban dwellers are largely educated with a significant presence on several social media platforms [41]. In some previous studies, several authors have reported negative association between social media exposure and the acceptance of COVID-19 vaccines [66,67,68,69,70,71,72], while others reported positive association [73,74,75,76].

Moreover, Murphy et al. [77], Lin et al. [55] and Yoda and Katsuyama [78] previously found that urban residents have a lower probability of willingness to be vaccinated against COVID-19, while a higher likelihood was reported by Abedin et al. [42] and Khubchandani et al. [79].

The findings also show high compliance with standard COVID-19 protective methods of washing hands after returning home from public places and the wearing of masks in public places. Moreover, the COVID-19 prevention compliance index significantly increased odds of being vaccinated against COVID-19. This is also an indication that compliance with protective methods against COVID-19 is directly associated with the decision to be vaccinated. This finding is in alignment with that of Abedin et al. [42] and Urrunaga-Pastor [80]. The finding also sheds some insights on the complementary perceptions of vaccination and engagement with other preventive methods.

Risk perception has been considered as the heart of compliance with disease prevention. The findings from the study revealed that the worry of COVID-19 sickness and the perceived threat it poses to households’ finances significantly increased the odds of willing to be vaccinated. Similarly, those households that needed medical services also had higher log odds of willing to get vaccinated. These findings are in alignment with expectations as reported in some recent studies. Specifically, the perception of COVID-19 risk or the extent of vulnerability was reported to influence the acceptability of vaccines by Ehde et al. [81], Guidry et al. [43], Alqudeimat et al. [57], Wong et al. [82] and Detoc et al. [83].

The results showed that the parameters of education and gender were not statistically significant in influencing willingness to be vaccinated against COVID-19. However, age showed statistical significance with a positive sign. This is in alignment with some previous studies that found age as a factor that increases the probability of willing to be vaccinated against COVID-19 [38,39,40,41] but contrary to the findings of some studies [42,43,44].

## 5. Conclusions

Controlling the spread of COVID-19 in Nigeria is a development objective with timelessly invaluable dividends. This is due to peculiar weaknesses of the healthcare delivery system, economic fragility and high population pressure. More importantly, Nigeria exhibits significant diversities that can subject it to significant epidemiological vulnerability given the pathological characteristics of COVID-19. Healthcare policy makers have therefore taken strict measures to guarantee economic sustainability in the country by preventing the spread of the virus. The economic implications of the efforts aimed at containing the virus can be evaluated from the willingness of the people to be vaccinated against COVID-19. The findings of this study are therefore beacons that point at specific interventions for the successful implementation of COVID-19 vaccinations against the raging virus.

Although the acceptance rate of COVID-19 is high, it may not be high enough until everyone complies when dealing with a pandemic of high severity and transmission rate like COVID-19. There is therefore the need to ensure proper interventions in ensuring that individuals who have shown unwilling to be vaccinated against COVID-19 are positively orientated to ensure a positive response. This may take significant advocacy interventions to diffuse ongoing misconceptions that are promoting vaccine hesitancy. Such efforts should focus on urban households and those from southern Nigeria through social media and other informal mechanisms, although rural areas and northern Nigeria cannot be completely neglected. It is also important to note that although the age of respondents was found to increase the log odds of the willingness to be vaccinated against COVID-19, the margin of impacts is very small. In addition, the perception of health and financial risks that are associated with COVID-19 infection constitutes a significant factor that influences willingness to be vaccinated against COVID-19. This points to the need for adequate interventions to enhance individuals’ capability to properly evaluate their levels of COVID-19 risk. Such interventions should also target old people to enhance their willingness to be vaccinated against COVID-19.

## Figures and Tables

**Table 1:** Coding of Selected Variables for Logit Regression Analysis.

Variable	Mean	Std. Dev.	Min	Max
Willingness to take COVID vaccine (yes = 1, 0 otherwise)	0.853	0.354	0	1
Preventive compliance index—computed with Principal Component Analysis (PCA) with hand washing and wearing of mask variables	0.000	1.206	−1.75	1.14
Number of events attended	2.386	1.716	0	5
Needed medical services (yes = 1, 0 otherwise)	0.437	0.496	0	1
Male household head (yes = 1, 0 otherwise)	0.819	0.385	0	1
Age of respondents (years)	50.202	14.699	19	99
**Sector and Geopolitical Zones**				
Urban resident (yes = 1, 0 otherwise)	0.392	0.488	0	1
North Central zone (yes = 1, 0 otherwise)—reference group				
North East zone (yes = 1, 0 otherwise)	0.183	0.387	0	1
North West zone (yes = 1, 0 otherwise)	0.157	0.364	0	1
South East zone (yes = 1, 0 otherwise)	0.179	0.384	0	1
South South zone (yes = 1, 0 otherwise)	0.128	0.334	0	1
South West zone (yes = 1, 0 otherwise)	0.192	0.394	0	1
**Worriedness of Household Members Contracting COVID-19**				
Very worried (yes = 1, 0 otherwise)—reference group	0.639	0.480	0	1
Somewhat worried (yes = 1, 0 otherwise)	0.087	0.282	0	1
Not too worried (yes = 1, 0 otherwise)	0.085	0.279	0	1
Not worried at all (yes = 1, 0 otherwise)	0.189	0.391	0	1
**COVID-19 Is a Substantial Threat to Household’s Finance**				
Substantial threat (yes = 1, 0 otherwise)—reference group	0.665	0.472	0	1
Moderate threat (yes = 1, 0 otherwise)	0.162	0.369	0	1
Not much of a threat (yes = 1, 0 otherwise)	0.084	0.278	0	1
Not a threat at all (yes = 1, 0 otherwise)	0.089	0.285	0	1
**Educational Qualifications**				
No education (yes = 1, 0 otherwise)—reference group	0.231	0.422	0	1
Primary education (yes = 1, 0 otherwise)	0.211	0.408	0	1
Modern/Secondary education (yes = 1, 0 otherwise)	0.315	0.465	0	1
Tertiary education (yes = 1, 0 otherwise)	0.215	0.411	0	1
Vocational education (yes = 1, 0 otherwise)	0.028	0.166	0	1

**Table 2:** Distribution of Respondents’ Willingness to be Vaccinated against COVID-19.

Zone	Not Willing	% of Total	Willing	% of Total	Total
North Central	30	10.99	243	89.01	273
North East	12	3.86	299	96.14	311
North West	19	7.12	248	92.88	267
South East	86	28.20	219	71.80	305
South South	47	21.66	170	78.34	217
South West	56	17.13	271	82.87	327
**Sector**					
Rural	126	12.20	907	87.80	1033
Urban	124	18.59	543	81.41	667
**Education Level**					
None	42	10.69	351	89.31	393
Primary	71	19.78	288	80.22	359
Modern/Secondary	59	11.03	476	88.97	535
Tertiary	73	20.00	292	80.00	365
Vocational	5	10.42	43	89.58	48
**Gender**					
Female	60	19.54	247	80.46	307
Male	190	13.64	1203	86.36	1393
**Age**					
<30	16	16.16	83	83.84	99
30 < 40	52	15.20	290	84.80	342
40 < 50	60	13.79	375	86.21	435
50 < 60	50	12.99	335	87.01	385
≥60	72	16.40	367	83.60	439
**Hand Washing**					
No	74	18.36	329	81.64	403
Yes	176	13.57	1121	86.43	1297
**Mask Wearing**					
No	54	16.02	283	83.98	337
Yes	196	14.38	1167	85.62	1363
**Needed Medical Services**					
No	173	18.08	784	81.92	957
Yes	77	10.36	666	89.64	743
**No. of Gatherings Attended**					
None	35	19.13	148	80.87	183
One	85	16.1	443	83.9	528
Two	48	16.61	241	83.39	289
Three	32	14.55	188	85.45	220
Four	9	8.257	100	91.74	109
Five	41	11.05	330	88.95	371
**Total**	250	14.71	1450	85.29	1700

**Table 3:** Respondents’ Perceptions on Being Worried and Threats Posed by COVID-19.

Zone	Not Willing	% of Total	Willing	% of Total	Total
Very worried	111	10.21	976	89.79	1087
Somewhat worried	13	8.78	135	91.22	148
Not too worried	37	25.69	107	74.31	144
Not worried at all	89	27.73	232	72.27	321
Substantial threat	133	11.77	997	88.23	1130
Moderate threat	45	16.30	231	83.70	276
Not much of a threat	31	21.68	112	78.32	143
Not a threat at all	41	27.15	110	72.85	151
Total	250	14.71	1450	85.29	1700

**Table 4:** Logit Regression Results of the Determinants of Willingness to Take COVID-19 Vaccines.

Variables	Coefficient	Z Statistics	Odds Ratio	Z Statistics	dy/dx	Z Statistics	VIF
Urban Areas	−0.351 **	−2.12	0.704 **	−2.12	−0.035 **	−2.05	1.29
Compliance Index	0.130 **	2.06	1.139 **	2.06	0.013 **	2.07	1.10
No. of events attended	−0.001	−0.03	0.999	−0.03	0.000	−0.03	1.19
Needed Medical Services	0.319 **	1.97	1.376 **	1.97	0.030 **	2.00	1.11
Male Headed Households	0.257	1.41	1.293	1.41	0.027	1.33	1.10
Age of Household Heads	0.014 **	2.48	1.014 **	2.48	0.001 **	2.48	1.18
Primary education	−0.214	−0.92	0.808	−0.92	−0.022	−0.88	1.63
Modern/Secondary education	0.419	1.69	1.520	1.69	0.038	1.8	1.94
Tertiary education	−0.369	−1.53	0.691	−1.53	−0.039	−1.42	1.76
Vocational/Diploma Certificate	0.130	0.24	1.139	0.24	0.012	0.25	1.13
North East	0.848 **	2.31	2.334 **	2.31	0.067 ***	2.95	1.90
North West	0.221	0.68	1.247	0.68	0.020	0.72	1.87
South East	−1.092 ***	−4.33	0.336 ***	−4.33	−0.139 ***	−3.47	1.91
South South	−0.831 ***	−3.10	0.436 ***	−3.10	−0.102 **	−2.53	1.61
South West	−0.407	−1.5	0.665	−1.5	−0.044	−1.36	2.14
Somewhat worried about having COVID	−0.079	−0.39	0.924	−0.39	−0.008	−0.38	1.13
Not too worried about having COVID	−0.489 **	−1.99	0.613 **	−1.99	−0.055	−1.73	1.11
Not worried at all about having COVID	−0.757 ***	−3.28	0.469 ***	−3.28	−0.093 ***	−2.68	1.11
COVID is moderate threat to finance	0.413	1.29	1.511	1.29	0.035	1.48	1.09
COVID not much of a threat to finance	−0.652 ***	−2.71	0.521 ***	−2.71	−0.078 **	−2.26	1.17
COVID not a threat at all to finance	−0.686 ***	−3.78	0.504 ***	−3.78	−0.079 ***	−3.24	1.28
Constant	1.666 ***	3.77	5.291 ***	3.77			
Number of observation	1700		1700				
LR chi2(21)	191.41 ***		191.41 ***				
Pseudo R2	0.1348		0.1348				
Variance Inflation Factor	1.42						

Note: ***—Statistically significant at 1%; **—Statistically significant at 5%.

## Data Availability

The data used for this study are in the public domain. The data were downloaded from https://microdata.worldbank.org/index.php/catalog/3712/study-description#metadata-data_access (accessed on 15 June 2021).
